# A decrease in physiological arousal accompanied by stable behavioral performance reflects task habituation

**DOI:** 10.3389/fnins.2022.876807

**Published:** 2022-07-22

**Authors:** Andreea Micula, Jerker Rönnberg, Yue Zhang, Elaine Hoi Ning Ng

**Affiliations:** ^1^Department of Behavioral Sciences and Learning, Linnaeus Centre HEAD, Swedish Institute for Disability Research, Linköping University, Linköping, Sweden; ^2^Oticon A/S, Smørum, Denmark; ^3^Oticon Medical, Nice, France

**Keywords:** pupil baseline, task engagement, task habituation, free recall, working memory capacity, background noise, hearing loss

## Abstract

Despite the evidence of a positive relationship between task demands and listening effort, the Framework for Understanding Effortful Listening (FUEL) highlights the important role of arousal on an individual’s choice to engage in challenging listening tasks. Previous studies have interpreted physiological responses in conjunction with behavioral responses as markers of task engagement. The aim of the current study was to investigate the effect of potential changes in physiological arousal, indexed by the pupil baseline, on task engagement over the course of an auditory recall test. Furthermore, the aim was to investigate whether working memory (WM) capacity and the signal-to-noise ratio (SNR) at which the test was conducted had an effect on changes in arousal. Twenty-one adult hearing aid users with mild to moderately severe symmetrical sensorineural hearing loss were included. The pupil baseline was measured during the Sentence-final Word Identification and Recall (SWIR) test, which was administered in a background noise composed of sixteen talkers. The Reading Span (RS) test was used as a measure of WM capacity. The findings showed that the pupil baseline decreased over the course of the SWIR test. However, recall performance remained stable, indicating that the participants maintained the necessary engagement level required to perform the task. These findings were interpreted as a decline in arousal as a result of task habituation. There was no effect of WM capacity or individual SNR level on the change in pupil baseline over time. A significant interaction was found between WM capacity and SNR level on the overall mean pupil baseline. Individuals with higher WM capacity exhibited an overall larger mean pupil baseline at low SNR levels compared to individuals with poorer WM capacity. This may be related to the ability of individuals with higher WM capacity to perform better than individual with poorer WM capacity in challenging listening conditions.

## Introduction

Hearing aid users often experience that listening is tiring, effortful or stressful, even when the speech is loud enough to be understood (Pichora-Fuller et al., 2016). This experience may lead to withdrawal from social interactions, which may contribute to a feeling of social isolation, depression and generally lower life quality (Arlinger, 2003; Keidser et al., 2015; Pichora-Fuller et al., 2016). Listening effort has been defined as the “the deliberate allocation of mental resources to overcome obstacles in goal pursuit when carrying out a listening task” (Pichora-Fuller et al., 2016). A combination of behavioral and physiological measures has been used in order to investigate listening effort and cognitive resource allocation during listening tasks. Specifically, such studies often combine pupillometry with speech recognition or auditory recall test in order to investigate the effects of various manipulations, such as background noise and hearing aid signal processing (Wendt et al., 2017; Ohlenforst et al., 2017, 2018; Zekveld et al., 2018; Bönitz et al., 2021; Micula et al., 2021). Additionally, pupillometry in combination with behavioral measures has also been used to track levels of arousal, in order to investigate the effects of time-on-task on engagement and fatigue (Hopstaken et al., 2015; Alhanbali et al., 2020; Ayasse and Wingfield, 2020).

In the current study, pupillometry is used in order to investigate potential fluctuations in arousal and their effect on behavioral performance during an auditory recall task. Thus, the combination of physiological and behavioral responses is considered to be a marker of task engagement. Arousal has been described as a “fundamental property of behavior,” which is closely linked to phenomena such as attention, motivation, stress, anxiety, and sleep (Aston-Jones and Cohen, 2005). The locus coeruleus norepinephrine (LC-NE) system has been associated with regulation of physiological arousal. Thus, low LC-NE activity is considered to reflect low levels of arousal and vice versa (Aston-Jones and Cohen, 2005; McGarrigle et al., 2017; Joshi and Gold, 2020). According to the Framework for Understanding Effortful Listening (FUEL), which Pichora-Fuller et al. (2016) developed based on the Capacity Model of Attention (Kahneman, 1973), the cognitive capacity that can be allocated to a task is limited and is modulated by levels of arousal. The levels of arousal and how cognitive resources are allocated depend on the listener’s evaluation of task demands, which may in turn be affected by factors such as displeasure with the task. The levels of arousal may also be influenced by input-related factors, such as background noise or decreased speech signal quality due to hearing loss. Pichora-Fuller et al. (2016) highlight the importance of understanding the role of arousal in the listener’s choice to engage in effortful listening. Despite the evidence that there is a positive relationship between task demands and listening effort (Ohlenforst et al., 2018), the FUEL also points out that this relationship is more complex. That is, arousal and motivation may determine whether a listener continues to expend effort and engage in a listening task, even if the demands do not exceed the individual’s cognitive capacity (see also Herrmann and Johnsrude, 2020). Lemke and Besser (2016) define task engagement as the “readiness to invest resources to accomplish a task goal.” Disengagement implies temporarily abandoning a task (Lemke and Besser, 2016). This definition has been adopted in the FUEL.

Pupillary responses have been linked to the LC_NE system, which is activated in response to arousal, stress, anxiety, as well as cognitive processes such as cognitive resource allocation and memory formation. Typically, pupillary responses are divided into two types, task-evoked and baseline pupillary responses. Task-evoked pupillary responses are transient responses triggered by specific task stimuli. Baseline pupillary responses reflect the overall brain state, which may affect arousal or attention over relatively long time intervals (Joshi and Gold, 2020). While the task-evoked pupillary responses have been widely used to investigate cognitive resource allocation during a task (Zekveld et al., 2018), baseline pupillary responses have been predominantly used as an index of arousal and task engagement (Hopstaken et al., 2015; Winn et al., 2018; Alhanbali et al., 2020; Ayasse and Wingfield, 2020). The baseline pupil diameter is considered to reflect the levels of arousal, such that increasing baseline pupillary responses are considered to reflect heightened levels of arousal (Aston-Jones and Cohen, 2005; Joshi and Gold, 2020). The baseline pupillary response is usually measured immediately prior to the task stimulus (Winn et al., 2018).

Since baseline pupillary responses have been linked to arousal and arousal is considered to have an effect on a person’s choice to engage in a listening task, pupillometry has been used as an indirect index of task engagement. Previous studies have found a link between lower baseline pupillary responses and decreased behavioral performance. In this case, baseline pupillary responses were considered to reflect a decrease in arousal related to disengagement from the task or the development of fatigue in both individuals with normal hearing (Hopstaken et al., 2015; Alhanbali et al., 2020) and individuals with varying degrees of hearing loss (Alhanbali et al., 2020). Interestingly, Ayasse and Wingfield (2020) found a different relationship between baseline pupillary responses over the course of an auditory sentence comprehension task and performance in the task. The participants’ hearing status ranged from normal hearing to moderate hearing loss. Their findings showed that although baseline pupil dilation decreased over the course of the task, and that the decrease was steeper for individuals with hearing loss, task performance increased slightly. These findings seem inconsistent with the interpretation of decreasing baseline pupillary responses as an index of lower task engagement, as task performance did not decrease. Individuals with poorer hearing exhibited higher baseline pupil dilations at the beginning of the test session, but ended up on a similar level as individuals with normal hearing toward the end of the test session. The authors suggest that individuals with poorer hearing may have experienced higher levels of anticipatory arousal due to the nature of the task, which relies on accurate speech perception. The level of anticipatory arousal seemed to gradually decrease with successful task performance. The findings of the study by Ayasse and Wingfield (2020) suggest that a decrease in bline pupillary responses does not necessarily reflect disengagement from the task or fatigue if the task performance remains constant or improves over time. In this case, baseline pupillary responses may instead reflect habituation. Task habituation refers to a decreased response as a consequence of repetition, without implying that a stimulus is not registered (Kahneman, 1973). As they habituate to a task, participants may re-evaluate the task demands to be lower, leading to a decrease in arousal. This is in agreement with the prediction made in the FUEL regarding the effect of task demands evaluation on arousal (Pichora-Fuller et al., 2016).

The Sentence-final Word Identification and Recall (SWIR) test is an auditory recall test designed to measure the effect of hearing aid signal processing on recall performance of highly audible speech heard in background noise. The SWIR test is typically conducted at an individualized SNR level that results in approximately 95% correct word recognition (Ng et al., 2013, 2015). This level is chosen in order to ensure that differences in SWIR test recall performance are due to differences in cognitive resource allocation rather than audibility in various conditions. In recent studies, the SWIR test has been combined with pupillometry in order to obtain behavioral and physiological indices of how various manipulations, such as noise reduction in hearing aids or task difficulty, affect cognitive resource allocation and listening effort (Bönitz et al., 2021; Micula et al., 2021). The SWIR test is a lengthy test, and the recall task and background noise make it cognitively demanding. Listeners with hearing loss, who may be more prone to experiencing increased listening effort (Pichora-Fuller et al., 2016), are often included in studies using the SWIR test (Ng et al., 2013, 2015; Micula et al., 2020, 2021). It could be speculated that at some point during a test session arousal may decrease due to the factors illustrated in the FUEL, such as displeasure with the task. Consequently, task engagement may decrease over the course of the test session. If listeners disengage from the task, it could interfere with capturing the effect of various manipulations on cognitive resource allocation. Therefore, it is important to track how arousal, indexed by the baseline pupillary responses, alongside recall performance, vary over the course of the SWIR test. Based on previous studies (Hopstaken et al., 2015; Alhanbali et al., 2020; Ayasse and Wingfield, 2020), a decrease in the pupil baseline is expected in the SWIR test. The behavioral recall performance is essential in facilitating the interpretation of this expected decrease. If the decrease in pupil baseline is accompanied by a decrease in recall performance, it is considered to reflect disengagement from the task. If recall performance does not decrease, the decrease in pupil baseline is considered to indicate task habituation rather than disengagement.

Ayasse and Wingfield (2020) included age and pure-tone average (PTA) thresholds in their analyses in order to investigate whether these individual factors contributed to the change in baseline pupillary responses across trials. However, a group of participants with wider age and PTA range was included in their study and the test was conducted unaided. In the current paper, the test was conducted using hearing aids and the PTA and age ranges were more homogenous. Therefore, working memory (WM) capacity measured using the Reading Span (RS) test and the individual signal-to-noise ratio (SNR) at which the test was conducted were included. While both the RS test and the SWIR test tap into WM capacity, the former is visually presented and thus provides a measure that is not affected by hearing ability. Although the SNR level is individualized so as to equalize the task difficulty amongst participants (ca. 95% correct word recognition), the individual SNR level may provide information about the listeners’ inherent ability for speech recognition in noise.

## Aims

The current study investigated whether changes in the pupil baseline and recall performance over the course of the SWIR test reflect reduced task engagement (Hopstaken et al., 2015; Alhanbali et al., 2020), or task habituation (Ayasse and Wingfield, 2020). This has not been previously explored using the SWIR test. It is relevant to gain such insights about a test that is designed to evaluate cognitive resource allocation especially in individuals with hearing loss, who may be more prone to developing listening fatigue (Pichora-Fuller et al., 2016).

The first aim was to investigate potential fluctuations in arousal over the course of the SWIR test, indexed by changes in the pupil baseline. A decrease in pupil baseline is expected over the course of the task. If this decrease is accompanied by a decrease in behavioral recall performance, it is hypothesized that the decrease in arousal is linked to reduced task engagement (Hopstaken et al., 2015; Alhanbali et al., 2020). However, if recall performance does not decrease over the course of the task, it is hypothesized that the decrease in arousal is a result of habituation to the task (Ayasse and Wingfield, 2020).

The second aim was to investigate whether individual factors, namely WM capacity measured using the RS test and the SNR level at which the SWIR test was conducted, modulate the change in arousal levels, indexed by the pupil baseline over the course of the task. Based on evidence that individuals with higher WM capacity perform better in the SWIR test (Ng et al., 2013; Micula et al., 2020), it is expected that this group of participants will maintain a higher degree of task engagement throughout the test session than individuals with lower WM capacity.

## Materials and methods

### Participants

Twenty-one native Danish speakers (8 female, 13 male) with mild to moderately severe symmetrical sensorineural hearing loss participated in the current study. The mean age of the test participants was 58 years (*SD* = 11.3, range: 22–73). The wide age range is driven by the age gap of 20 years between the two youngest participants. It should be noted that no significant correlation was found between age and pupillary responses. The participants’ mean PTA at 0.5, 1, 2 and 4 kHz was 49.30 dB HL (*SD* = 11.44, range: 25.00–73.13). The test participants were experienced hearing aid users (minimum 1 year), had no history of eye surgery or disease and had normal or corrected to normal vision. The group of test participants in the current study was a subset of a group recruited from the database at Eriksholm Research Centre, Snekkersten, Denmark, for a larger study. Four participants were excluded from the larger study due to insufficient pupillary response data (see section “Pupillometry”) and one participant was excluded due to not completing the RS test. The test participants signed a written informed consent form, and the study was conducted in accordance with the Declaration of Helsinki. The study was exempted from ethical application by the Science Ethics Committee for the Capital Region of Denmark (journal no. H-20028542).

### Assessment tools

#### The sentence-final word identification and recall test

A Danish version of the SWIR test (Lunner et al., 2016), composed of sentences from the Danish Hearing In Noise Test (HINT) (Nielsen and Dau, 2011), was used in the current study. The SWIR test was similar to the one used by Lunner et al. (2016), but it was modified by re-arranging the sentences into new lists. The task of the SWIR test is to listen to lists of seven sentences, repeat only the last word immediately after the sentence and at the end of the list, signaled by a beep tone, recall as many of the repeated words as possible regardless of order and with no time limit (free recall). The order of the lists was randomized for each participant. Twenty-eight lists of seven sentences were administered, which will be referred to as blocks. The recall performance was scored as the percentage of correctly recalled words per block. Misperceived words that were correctly recalled were included in the score.

#### Pupillometry

**FIGURE 1:**
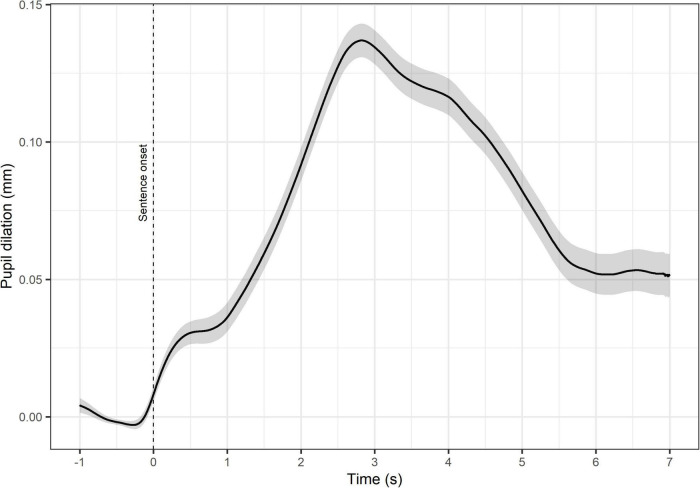
Time course of the pupillary response averaged during a time interval ranging from the background noise 1 s prior to the sentence onset (dotted line) and up until the beginning of the background noise corresponding to the following sentence. The pupillary response prior to the sentence onset is defined as sentence baseline. The shaded area represents the confidence intervals at the 95% level.

Pupillary responses were recorded using the Tobii Spectrum Eye Tracker (Tobii Technology AB 2019) at a sampling frequency of 1,200 Hz. Per default, data from the right eye was analyzed, unless more data was available for the left eye of a test participant. A 1s-long sliding window (1,200 samples) was used for detecting blinks. Samples with pupil dilation below the minimum threshold, set to 3 SDs below the mean pupil size within the sliding window, were considered as blinks. Blinks were removed from the signal, including 77 samples (64 ms) before and the 181 samples (151 ms) after (Siegle et al., 2008). After blink removal, linear interpolation was implemented to replace missing values. Sentences with less than 60% of valid data were discarded. Participants for whom more than 15% of the sentences were missing were excluded from the analyses. On average, 1.65% of the sentences were discarded per participant. Figure 1 shows the time course of the pupillary response averaged during an interval starting from 1 s prior to the SWIR test sentence onset (sentence baseline) and until the beginning of the background noise corresponding to the following sentence.

**FIGURE 2:**
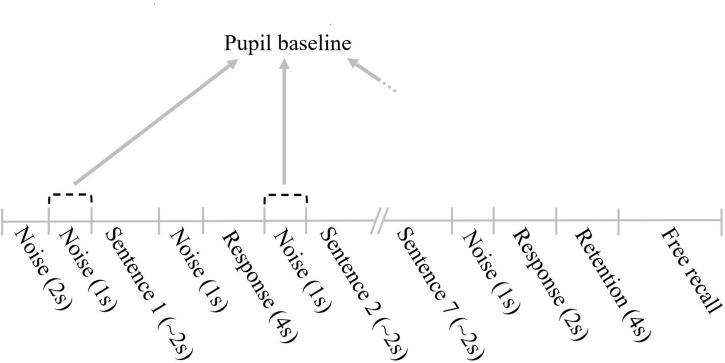
Illustration of the structure of a SWIR test block and the method of calculating the pupil baseline. The duration of the different phases of the block are indicated in seconds (s).

The sentence baseline pupillary response was defined as the mean pupil dilation during a time interval of 1 s prior to the beginning of a sentence (Figure 1). This way of calculating the sentence baseline pupillary responses has been used in previous studies combining the SWIR test and pupillometry (Bönitz et al., 2021; Micula et al., 2021). These studies have also shown that the sentence baseline pupillary responses tend to increase over the course of several SWIR test sentences, reflecting the resources allocated to storing an increasing number of items for later recall. In order to minimize this memory effect, the sentence baseline pupillary response of each sentence in a block were averaged. This averaged value is referred to as the pupil baseline and is considered to index arousal over the course of the blocks. Figure 2 illustrates the structure of a SWIR test block and how the pupil baseline is calculated (see further details in Test Conditions and Set-up).

#### Reading span test

The RS test is commonly used as a measure of WM capacity, since it requires simultaneous processing and storage of information (Daneman and Carpenter, 1980; Rönnberg et al., 1989). A short version (Micula et al., 2020, 2021, in review) of the Danish RS test (Borch Petersen et al., 2016) was used. All sentences are composed of three words. These are arranged in lists of three-, four- and five sentences. Before the test, one list of three sentences was administered for procedural training. Twenty-four sentences, divided in two lists of each length, were presented on a laptop screen in ascending order. Half of the sentences were sensible, and half were absurd. The task involves reading the sentences and verbally indicating after each sentence whether it was sensible or absurd. At the end of each list, the test participants were asked to either recall the first or the last words of each sentence. The RS test score calculated as the percentage of correctly recalled words, regardless of recall order (Borch Petersen et al., 2016).

### Test conditions and set-up

The SWIR test sentences were presented in a background noise composed of 16-talker babble held constant at 70 dB SPL. The level of the target sentences was individualized for each participant to an SNR level estimated to result in 95% word recognition (see Procedure). The background noise started 3 s prior the first sentence of the block and 1 s prior to the remaining six sentences. The background noise stopped 1 s after the end of each sentence. Between the end of the background noise of one sentence and the beginning of the background noise of the following sentence there was a silent interval with a duration of 4 s (see response in Figure 2), during which the participants repeated the last word. The response interval after the last sentence was shortened to 2 s, due to the following retention interval with a duration of 4 s. During the retention interval the participants waited for the beep tone cuing the free recall phase (see Retention and Free recall in Figure 2). When the participants indicated that they could not recall additional words, the experimenter selected the list for the following block, which resulted in a short break of approximately 5–10 s between blocks.

The test participants were seated in a soundproof anechoic chamber. The target sentences were presented from a loudspeaker placed at 0°. The background noise was presented from four loudspeakers placed at ± 112.5° and ± 157.5°. These loudspeakers played recordings of two male and two female native Danish speakers reading different newspaper passages. The loudspeakers were placed a distance of 1.2 m from the participant. The eye-tracker was placed 60 cm in front of the test participant.

Four test conditions were included for the purpose of the larger study: two models of hearing aids (Oticon OpnS1™ and Oticon More™ mini-Receiver-in-the-ear) with noise reduction activated and deactivated. Preliminary analysis found no significant differences between these conditions. The hearing aid conditions were pooled for the analysis, since investigating differences between them was not within the scope of the current study.

### Procedure

Each participant took part in a single test visit with an overall duration of approximately two h. The SWIR test was administered first, followed by the RS test. In order to estimate the individual SNR required for the SWIR test, the HINT was administered using a modified procedure in order to achieve 80% speech intelligibility. Both the target sentences and the background noise were presented at 70 dB SPL at the beginning of the HINT. The level of the background noise remained constant, while the level of the target speech fluctuated based on the participants’ responses. If a sentence was correctly repeated, the SNR was decreased by 0.8 dB. After incorrect repetition, the SNR was increased by 3.2 dB. For the first five sentences the step size was double. The SNR resulting from the HINT test was used as a starting point for the SWIR test training. It has been shown that the SNR level at which participants reach 80% speech intelligibility in the HINT roughly corresponds to 95% word recognition in the SWIR test (Lunner et al., 2016), since correctly repeating all the words in a sentence (HINT) is more demanding than repeating a single word (the last word of a SWIR test sentence). Four SWIR test training lists of seven sentences were administered in order verify whether 95% word recognition was achieved (Lunner et al., 2016; Micula et al., 2020, 2021). If six or seven of the last words of each sentence were repeated correctly (86–100%), the SNR obtained from the HINT was not changed. If four or five words were repeated correctly, the SNR was increased by 1 dB. If zero to three words were repeated correctly, the SNR was increased by 2 dB. The SNR obtained after the fourth training list was used for the remainder of the SWIR test. The participants were offered a break of approximately 10 min half-way through the SWIR test, and they had the opportunity to ask for additional breaks if needed. Excluding breaks, the duration of the SWIR test was about 1 h.

### Statistical analysis

Linear mixed effects models were built using the *lmer* function from the *lme4* package (Bates et al., 2021, 2015) in R version 4.0.2 (R Core Team, 2020). Two identical models were built, except for the outcome variables. In the first model the outcome variable was the pupil baseline, while in the second model the outcome variable was the recall performance in the SWIR test. Block, RS test score and SNR were defined as continuous variables and were centered using the *scale* function before being entered as fixed effects in the models, including the three-way interaction between them. Participant was added as a random effect, so that by-participant random intercepts and random slopes for Block, RS test score and SNR were included. However, both models were affected by convergence issues and singular fit warnings, suggesting that they were over-parameterized. In order to avoid over-fitting, the *step* function was used, which automatically performs backward elimination of non-significant effects from the random structure first, followed by backward elimination of non-significant fixed effects.

## Results

The mean RS test score was 41.72% (*SD* = 11.99, range: 25–75) and the mean SNR was 6.65 dB (*SD* = 3.08, range: 2.60–13.4), both being comparable to the values reported by Ng et al. (2015). The mean recall performance was 58.10% (*SD* = 18.31, range: 14.28–100), which is very similar to the recall performance obtained in the studies by Micula et al. (2020, 2021).

Block, RS test score and SNR, as well as the interaction between RS test score and SNR remained as fixed effects in the model with pupil baseline as the outcome after using the *step* function. Additionally, the random slopes for Block remained. For the model with recall performance as an outcome, only RS test score remained as a fixed effect, as well as the random slopes for Block, after applying the *step* function. An alternative method of building the models, by manually adding the fixed effects and interactions one at the time (similarly to Ayasse and Wingfield’s procedure), did not change the outcomes.

The output of the final model including the pupil baseline as the outcome variable is shown in Table 1. The conditional *R*^2^ indicate that the fixed and random effects in the model accounted for 94.6% of the variance and the marginal *R*^2^ indicated that the fixed alone accounted for 45.8% of the variance (Nakagawa and Schielzeth, 2013). The variance inflation factor was obtained for each variable in the model, showing that multicollinearity was low, as the highest value was 1.3.

**TABLE 1:** Output summary of the linear mixed effects model with baseline pupil dilation as the outcome variable.

	β (95% CI)	*p*
		
Intercept	3.48 (3.29, 3.68)	<0.001*
Block	−0.01 (−0.01, −0.00)	0.01*
RS test score	0.01 (−0.01, 0.02)	0.43
SNR	−0.09 (−0.14, −0.03)	0.01*
RS test score * SNR	−0.01 (−0.02, −0.00)	0.02*

The β-coefficient indicates the slope of the regression line for each fixed effect. The confidence intervals (CI) at the 95% level are shown in brackets. *p < 0.05.

### The effect of arousal on task engagement

The first aim of this study was to investigate whether changes in arousal, indexed by the pupil baseline, had an effect on task engagement.

**FIGURE 3:**
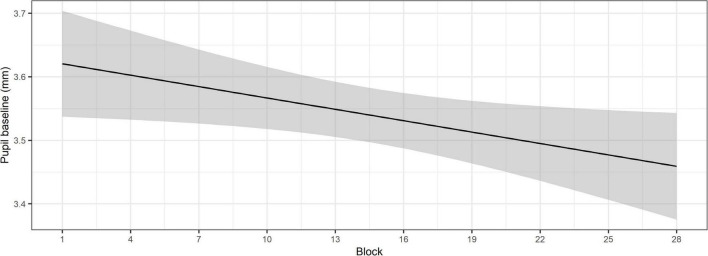
Change in pupil baseline over the course of the SWIR test blocks. The shaded area shows the confidence intervals at the 95%-level.

The ANOVA (Type II Wald χ^2^-tests) conducted on the model with pupil baseline as the outcome showed that there was a significant main effect of Block, χ^2^(1) = 9.44, *p* = 0.002, indicating that pupil baseline decreased across blocks. The significant main effect of Block is shown in Figure 3.

**FIGURE 4:**
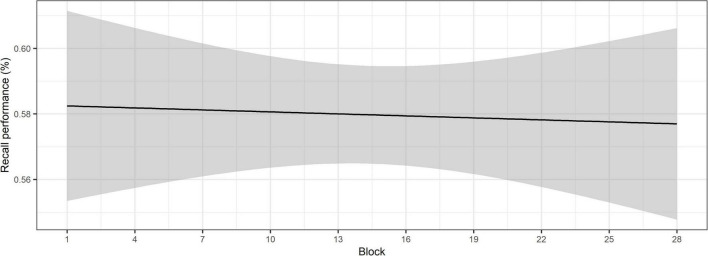
Recall performance over the course of the SWIR test blocks. The shaded area shows the confidence intervals at the 95%-level.

The ANOVA of the model with recall performance as the outcome showed that there was a significant main effect of RS test score on recall performance, χ^2^(1) = 4.89, *p* = 0.027. Higher RS test scores were associated with better recall performance. This relationship between WM capacity and recall performance in the SWIR test has been well-established in findings from previous papers (Ng et al., 2013; Micula et al., 2020). The findings suggest that recall performance does not change over the course of the SWIR test, since the Block factor was eliminated from the model. Figure 4 supports this, illustrating that recall performance remains relatively stable over the course of the blocks.

Together these findings indicate that while physiological arousal, indexed by the pupil baseline, decreases over the course of the SWIR test, behavioral recall performance does not.

### The effects of working memory capacity and individual signal-to-noise ratio level on arousal

The second aim of this study was to investigate whether WM capacity and the individual SNR level at which the SWIR test was performed at had an effect on the changes in arousal over the course of the task.

**FIGURE 5:**
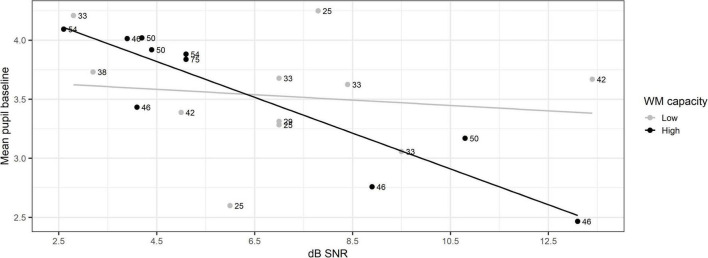
Mean pupil baseline as a function of Reading Span test score and signal-to noise ratio (SNR). The dots show the individual Reading Span test scores for the low working memory (WM) capacity group (light gray) and high WM capacity group (black). The regression lines were fitted for each group.

The model with pupil baseline as the outcome showed that there was a significant main effect of SNR, χ^2^(1) = 18.32, *p* < 0.001, indicating the pupil baseline decreases with increasing SNR. The main effect of RS test score was not significant, χ^2^(1) = 18.32, *p* = 0.06. Furthermore, the analysis showed that there was a significant interaction between RS test score and SNR, χ^2^(1) = 6.65, *p* = 0.001. Figure 5 shows the relationship between mean pupil baseline and SNR for each test participant. The individual RS test scores are indicated next to each individual data point. The individual data points were categorized based on median split into a group of 11 participants with low WM capacity (light gray dots) and a group of 10 participants with high WM capacity (black dots). The regression lines represent the relationship between mean pupil baseline and SNR for each group. Independent *t*-tests indicated that there were no significant differences between the groups in terms of age and PTA.

**FIGURE 6:**
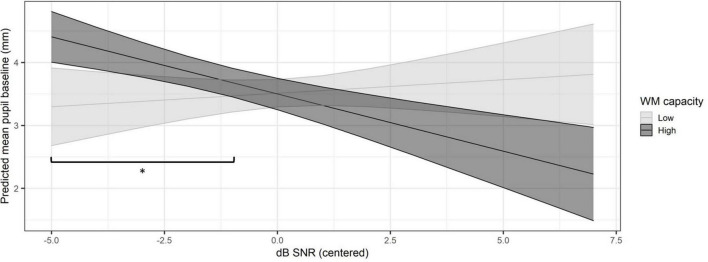
Illustration of the interaction between WM capacity and signal-to-noise ratio on the predicted mean pupil baseline. The low WM capacity group is represented in light gray and the high WM capacity group in dark gray. The shaded areas show the confidence intervals at the 95%-level. * Significant difference between the two WM capacity groups, *p* < 0.05.

In order to further explore the interaction between RS test score and SNR, additional *post hoc* analyses were conducted. For the *post hoc* analyses, the centered RS test scores used in the model were split in two groups based on the median. The mean of the centered values for each group was used. First, the simple slopes were explored using the *emtrends* function. The estimate of the simple slope of SNR was 0.01 (95% CI = −0.11, 0.12) for the low WM capacity group and −0.19 (−0.28, −0.10) for the high WM capacity group. The estimates and confidence intervals suggest that the simple slope of SNR is not significant for the low WM capacity group. However, for the high WM capacity group, the simple slope decreases significantly with increasing SNR. Furthermore, the pairwise comparisons of the simple slopes of the low and high WM capacity groups indicated that there was a significant difference between them (*p* = 0.026). Next, pairwise contrasts between WM capacity groups across SNR levels for the predicted values of mean pupil baseline were investigated using the *emmeans* function. All centered SNR values in the range −5 to 7 were tested in steps of 1. There was a significant difference between the two WM capacity groups for the centered SNR values ranging between −5 and −1 (*p* < 0.05). This finding suggests that individuals with higher WM capacity exhibited significantly higher mean pupil baseline values at low SNR levels compared to individuals with lower WM capacity. At higher SNR levels the difference in mean pupil baseline between the two groups was not significant. The interaction between SNR and RS test score is shown in Figure 6. The figure shows the predicted mean pupil baseline (based on the fitted model data) across SNR levels (centered) for the low and high WM capacity groups. Additionally, the SNR range for which the difference between the groups is significant is marked.

## Discussion

In the current study we investigated how arousal, indexed by the pupil baseline, and recall performance change over the course of the SWIR test blocks. The aim was to explore whether potential changes in arousal were associated with decreasing task engagement or task habituation. Furthermore, we investigated whether WM capacity and SNR had an effect on the change in arousal over the course of the blocks.

### The effect of arousal on task engagement

As hypothesized, the findings showed that the pupil baseline decreased over the course of the SWIR test blocks, reflecting a decrease in arousal levels. Since the recall performance did not significantly decrease over time, the findings are interpreted according to the task habituation hypothesis (Ayasse and Wingfield, 2020) rather than the declining task engagement hypothesis (Hopstaken et al., 2015; Alhanbali et al., 2020). This finding is particularly relevant, since the SWIR test is cognitively demanding, due to the recall task and the background noise. Under such conditions, arousal could be affected by factors highlighted in the FUEL, such as displeasure with the task and even development of fatigue. Thus, the analysis presented in the current paper may serve as a suitable verification of whether low task engagement or fatigue may be a confounding factor for data collected during long test visits. It should be noted, however, that the participants took at least one break during the test session, which decreases the likelihood of experiencing fatigue. This is usually preferred in studies using the SWIR test, so as to prevent fatigue from interfering with capturing cognitive resource allocation. Nonetheless, decreased behavioral performance may occur as a result of reduced task engagement without reaching the point of fatigue, due to factors such as lack of interest in the task, lack of motivation to invest the necessary resources to successfully perform the task or boredom amongst others (Pichora-Fuller et al., 2016).

Although changes in the level of arousal over the course of a task, reflected by a decrease in pupil baseline, are often associated with a decrease in task engagement, this does not seem to apply in the current study. In this case, supplementing the pupillary responses with behavioral responses has the advantage of facilitating the interpretation of the results. The stable recall performance over the course of the SWIR test suggests that the participants maintain a level of task engagement that allows allocating the necessary cognitive resources to the task. This is in line with the interpretation by Ayasse and Wingfield (2020), who attribute the decrease in baseline pupillary responses to a decrease in anticipatory arousal. The authors specify that the pupillary responses reflect the level of arousal at the time at which they are measured, in this case immediately prior to the sentence. Therefore, the term “anticipatory” is used to reflect the mental state at the point at which the baseline pupillary responses are measured. The decrease in anticipatory arousal was attributed to the task being evaluated as less challenging with familiarization to the procedure over time. This is in accordance with the FUEL, which identifies the listener’s evaluation of the task demands on capacity as one of the factors that modulates arousal (Pichora-Fuller et al., 2016). Based on the interpretation of findings from the study by Ayasse and Wingfield (2020), we suggest that as the participants habituate to the background noise and the procedure of the SWIR test, the perceived demands may decrease, resulting in lower arousal levels. Ayasse and Wingfield (2020) found that individuals with poorer hearing exhibited higher levels of anticipatory arousal at the beginning of the test session than individuals with normal hearing. In the present study, all test participants had hearing loss, which may partly account for the increased level of arousal at the beginning of the SWIR test.

Hearing aid users have been included in most previous studies in which the SWIR test was administered to investigate the effects of hearing aid signal processing on cognitive resource allocation (Ng et al., 2013, 2015; Micula et al., 2020, 2021). Such measures are particularly relevant for individuals with hearing loss, as they often report that listening is effortful (Pichora-Fuller et al., 2016; Alhanbali et al., 2019). Thus, these measures are essential, in order to learn how individuals with hearing loss allocate cognitive resources when listening to speech in background noise and how this allocation could be optimized in an attempt to reduce the experience of listening effort (Pichora-Fuller et al., 2016). Alhanbali et al. (2020) and Ayasse and Wingfield included participants with hearing loss alongside participants with normal hearing. In the former study, most participants with hearing loss performed the task using their own hearing aids, while in the latter the test was conducted unaided as none of the participants were regular hearing aid users. It should be noted that the task difficulty was equalized amongst participants in terms of audibility in both studies, as was the case in the current study. Hearing status was controlled for in the statistical analysis of both studies. Although Alhanbali et al. (2020) found evidence of disengagement from the task, Ayasse and Wingfield did not. These findings suggest that the ability to remain engaged is highly dependent on the task characteristics, regardless of hearing status. As mentioned previously, a decrease in task engagement may interfere with capturing the intended outcomes of the SWIR test. Offering breaks may be one way of preventing disengagement from the task. This is a choice that sets the current study apart from previous studies (Hopstaken et al., 2015; Alhanbali et al., 2020; Ayasse and Wingfield, 2020). However, the possibility of some participants developing fatigue or experiencing a deterioration in behavioral performance despite being offered breaks should not be disregarded, which may be seen as a potential limitation of the current study.

The Yerkes-Dodson Law (Yerkes and Dodson, 1908) is often referred to in studies investigating the relationship between arousal, task engagement and task performance (Hopstaken et al., 2015; Pichora-Fuller et al., 2016; Unsworth and Robison, 2017; Ayasse and Wingfield, 2020). According to the Yerkes-Dodson Law, the task engagement and task performance are lowest when the locus coeruleus activity is low (hypoarousal) or high (hyperarousal). The former condition is associated with states such as sleep and fatigue, while the latter is associated with states such as anxiety and stress. This relationship can be depicted as an inverted U-shaped curve. The level of arousal for optimal task engagement and performance lies between the extremes of hypo- and hyperarousal, at the highest point of the curve. Although the level of arousal was higher at the beginning of the SWIR test session, this level did not seem to be high enough to affect recall performance. Hence, this finding suggests that the levels of arousal of the participants in the current study fall within the optimal part of the curve.

### The effects of working memory capacity and individual signal-to-noise ratio level on arousal

There was no significant interaction between block and RS test scores or block and SNR levels, suggesting that these individual factors did not influence the decrease in pupil baseline over the course of the test session. These findings indicate that arousal decreases as a result of task habituation regardless of WM capacity or the individual SNR level at which the SWIR test is conducted. It was hypothesized that individuals with better WM capacity would be able to remain engaged in the SWIR test for a longer period of time compared to individuals with poorer WM capacity. The WM capacity may not play an important role for the decrease in arousal as a result of task habitation. The outcomes may have been different if the participants had reached the point of disengagement. The lack of effect of SNR on pupil baseline was likely due to the individualized level, which is estimated with the purpose of equalizing task difficulty amongst participants in terms of speech intelligibility. Further investigation is needed in order to determine whether the SNR level has an effect on changes in arousal during the SWIR test, by conducting the testing with a range of SNR levels for each participant rather than an individualized level.

A significant interaction between WM capacity and individual SNR level was found (Figure 6). This suggests that individuals with higher WM capacity exhibit a larger overall mean pupil baseline than individuals with lower WM capacity at low SNR levels. Zekveld et al. (2018) briefly mention in their review on pupillary responses to auditory stimuli that the loudness of a stimulus may evoke automatic attention effects. Automatic attention is considered to have an effect on cognitive resource allocation to a task according to the FUEL (Pichora-Fuller et al., 2016; Zekveld et al., 2018). Figure 5 shows that there is a wider range of SNR levels at which the SWIR test was conducted with participants categorized in the lower WM capacity group. However, most of the participants categorized in the high WM capacity group performed the test at lower SNR levels and only three participants at high SNR levels, with no participants in the middle SNR range. This indicates that participants with higher WM capacity are able to perform better than individuals with poorer WM capacity at lower SNR levels. Thus, the increased overall mean pupil baseline may be an index of automatic attention or other mechanisms that allow individuals with high WM capacity to maintain a stable level of cognitive resource allocation to the SWIR test and perform better even in more challenging listening conditions. Alternatively, the interaction may be a caused by the distribution of the data (Figure 5). The lower RS test scores were spread out across the range of SNR levels, while the higher RS test scores were mainly clustered at lower SNR levels with only three scores in the opposite end of the SNR level range. Further investigation is needed to understand the potential relationship between SNR and WM capacity and its effects on arousal and attention.

## Conclusion

The findings of the current study demonstrate that physiological arousal, indexed *via* the pupil baseline, decreases over the course of the SWIR test. A decrease in arousal is often associated with decreasing task engagement when it is accompanied by a decrease in behavioral performance over time (Hopstaken et al., 2015; Alhanbali et al., 2020). However, the present findings demonstrated that recall performance remained constant over the course of the test session. Thus, the decrease in arousal in this case was attributed to task habituation (Ayasse and Wingfield, 2020). This may result due to the participants re-evaluating the task demands, as they were becoming more familiar with the SWIR test procedure and the background noise. The decrease in pupil baseline was not influenced by RS test scores or the individual SNR level at which the test was conducted. This indicates that arousal decreases as a result of task habitation regardless of the WM capacity or the individual SNR level at which the SWIR test was performed.

Investigating fluctuations in arousal over the course of a test session and including behavioral responses to facilitate the interpretation of pupillary responses may provide valuable insights. Low task engagement may interfere with the outcomes of a test. Such insights may be valuable, particularly for the SWIR test, which is used to investigate the effects of various task demands on cognitive resource allocation.

## Data availability statement

The datasets presented in this article are not readily available because the data is part of a larger dataset collected at Eriksholm Research Centre. Requests to access the datasets should be directed to AM, andreea.micula@liu.se.

## Ethics statement

The studies involving human participants were reviewed and approved by the Science Ethics Committee for the Capital Region of Denmark. The patients/participants provided their written informed consent to participate in this study.

## Author contributions

AM designed and conducted the study, performed statistical analysis, and wrote the manuscript. YZ provided support with statistical analysis. EN, YZ, and JR designed the study and provided critical revision. All authors contributed to the article and approved the submitted version.

## Conflict of interest

EN and AM were employed by the Oticon A/S. YZ was employed by Oticon Medical. The remaining authors declare that the research was conducted in the absence of any commercial or financial relationships that could be construed as a potential conflict of interest.

## Publisher’s note

All claims expressed in this article are solely those of the authors and do not necessarily represent those of their affiliated organizations, or those of the publisher, the editors and the reviewers. Any product that may be evaluated in this article, or claim that may be made by its manufacturer, is not guaranteed or endorsed by the publisher.
